# Increased level of phosphorylated akt measured by chemiluminescence-linked immunosorbent assay is a predictor of poor prognosis in primary breast cancer overexpressing ErbB-2

**DOI:** 10.1186/bcr1015

**Published:** 2005-03-24

**Authors:** Jonas Cicenas, Patrick Urban, Vincent Vuaroqueaux, Martin Labuhn, Willy Küng, Edward Wight, Mark Mayhew, Urs Eppenberger, Serenella Eppenberger-Castori

**Affiliations:** 1 Stiftung Tumorbank Basel, Basel, Switzerland; 2 University Clinics, Department of Research, Molecular Tumor Biology, Basel, Switzerland; 3 OncoScore AG, Riehen, Switzerland; 4 University Clinics, Department of Gynecology, Basel, Switzerland; 5 University of Virginia, Department of Anatomy and Cell Biology, East Carolina School of Medicine, Charlottesville, Virginia, USA

## Abstract

**Introduction:**

Akt1, Akt2 and Akt3 kinases are downstream components of phosphoinositol 3-kinase derived signals from receptor tyrosine kinases, which influence cell growth, proliferation and survival. Akt2 overexpression and amplification have been described in breast, ovarian and pancreatic cancers. The present study was designed to investigate the prognostic significance of activated Akt in primary breast cancer and its association with other tumour biomarkers.

**Methods:**

Using a two-site chemiluminescence-linked immunosorbent assay, we measured the quantitative expression levels of total phosphorylated (P-S473) Akt (Akt1/Akt2/Akt3) on cytosol fractions obtained from fresh frozen tissue samples of 156 primary breast cancer patients.

**Results:**

Akt phosphorylation was not associated with nodal status or ErbB-2 protein expression levels. High levels of phosphorylated Akt correlated (*P *< 0.01) with poor prognosis, and the significance of this correlation increased (*P *< 0.001) in the subset of patients with ErbB-2 overexpressing tumours. In addition, phosphorylated Akt was found to be associated with mRNA expression levels of several proliferation markers (e.g. thymidylate synthase), measured using quantitative real-time RT-PCR.

**Conclusion:**

Our findings demonstrate that, in breast cancer patients, Akt activation is associated with tumour proliferation and poor prognosis, particularly in the subset of patients with ErbB2-overexpressing tumours.

## Introduction

Akt/protein kinase B (PKB) is a serine/threonine kinase that is involved in mediating various biological responses, such as inhibition of apoptosis and stimulation of cell proliferation (for review [1,2]). Three mammalian isoforms are currently known [1]: Akt1/PKBα, Akt2/PKBβ and Akt3/PKBγ. Akt1 was first discovered as a cellular homologue of the viral oncogene v-Akt, which causes leukaemia in mice [3] and is the predominant isoform in most tissues. High expression of Akt2 has been observed in insulin-responsive tissues, whereas Akt3 has been shown to be predominantly expressed in brain and testis [2].

Phosphoinositol-3-phosphate (PIP3) is a product of phosphoinositol 3-kinase enzymatic activity and has been shown to be a prerequisite lipid modulator of Akt activity [4]. PIP3 has been described as a downstream component of a wide range of receptors, including the c-Met receptor [5], the epidermal growth factor receptor family [6], fibroblast growth factor receptor [7], insulin growth factor receptor [8] and platelet-derived growth factor receptor [9]. In addition, Akt activity can be regulated by the PTEN tumour suppressor gene, which negatively regulates PIP3 levels (for review [10]). After PIP3 binding, Akt1 is activated by phosphorylation on two critical residues, namely threonine 308 (T308) and serine 473 (S473); similar activation residues (S472 and S474, respectively) are highly conserved in Akt2 and Akt3 (for review [1,2]). Several studies have found Akt2 to be amplified or overexpressed at the mRNA level in various tumour cell lines [11-13] and in a number of human malignancies, such as colon, pancreatic and breast cancers [14-16]. However, activation of Akt1, Akt2 and Akt3 by phosphorylation appears to be more clinically relevant than detection of Akt2 amplification or overexpression.

To date, several groups have investigated the phosphorylation of active Akt in breast, prostate, colon and pancreatic tumours by immunohistochemistry [14,17-22]. Under such conditions, phosphorylation structures may be disturbed by formalin fixation, rendering specific antigen sites inaccessible. Moreover, immunohistochemistry gives only semiquantitative results, limiting statistical analysis. Alternatively, enzyme immunoassays (EIAs) have the advantage that they yield highly reproducible and sensitive results of quantitative values.

In the present study we detected phosphorylated Akt (P-Akt) by means of a novel two-site chemiluminescence-linked immunoassay (CLISA) in fresh frozen primary tissue samples from 156 primary breast cancer patients. Because it was shown in previous immunohistochemistry studies that S473 P-Akt has prognostic significance [17-19], the aim of the present study was to measure levels of P-Akt continuously using CLISA and correlate these with survival and factors that are involved in tumourigenesis. Given that the antibody used in the reported immunohistochemistry studies recognized all Akt isoforms, we have developed an assay that allows specific quantitative detection of active Akt1, Akt2 and Akt3 when phosphorylated on their corresponding residues, namely S473, S472 and S474, respectively.

## Materials and methods

### Tumor and patient characteristics

Fresh material obtained during surgery was kept on ice and examined by a pathologist. Representative specimens with more than 60% tumour cells were sent to the Stiftung Tumorbank Basel (STB), immediately shock frozen and cryopreserved (-80°C). All activities of the STB are in accordance with an official Swiss permit, which guarantees patient confidentiality and respects ethical issues. For the present study, 156 samples of primary breast tumours were selected. Those samples overexpressing ErbB-2 (>500 U/mg total protein) were selected, based on ErbB-2 protein expression levels routinely detected using EIAs at the time of surgery by the STB [23]. EIA ErbB-2 positive samples correlate strongly with DAKO 3+ and with ErbB-2 amplification detected by fluorescent *in situ *hybridization (FISH; data not shown).

All patients underwent primary surgery before January 1996. Sixty-seven patients (43%) experienced disease recurrence within the median follow-up time of 57 months (range 27–88 months). Sixty-six patients (42%) were node negative, and 90 (58%) were node positive. Forty tumours (26%) were oestrogen receptor (ER)-α negative. Ninety-five patients (61%) had ErbB-2-negative (<500 U/mg total protein) and 61 patients (39%) had ErbB-2 positive tumours [23]. None of the patients received neoadjuvant therapy. Patient and tumour characteristics are summarized in Table 1.

### Cell lines and tissue culture

MCF-7 breast cancer cells were cultured in IMEM-ZO (improved minimal essential medium with zinc option) supplemented with 5% foetal bovine serum, l-glutamine and antibiotics (penicillin/streptomycin) at 37°C in a 5% carbon dioxide incubator. For the phospho-standard preparation, subconfluent MCF-7 cells were serum starved for 48 hours in serum-free media, and were treated with NaF and Na_3_VO_4 _for 1 hour, and then with 10% foetal bovine serum for 10 min. Cells were lysed for 5 min on ice in EB lysis buffer (20 mmol/l Tris-HCl [pH 7.4], 0.5 mol/l NaCl, 10 mmol/l EDTA, 1% Triton X100, 20 mmol/l NaF, 20 mmol/l glycerophosphate, 2 mmol/l Na_3_VO_4_, proteinase inhibitor cocktail [Roche, Indianapolis, IN, USA]), centrifuged at 20,000 *g *for 5 min and supernatant was stored at -80°C.

### Measurement of oestrogen receptor, progesterone receptor and ErbB-2 protein levels in tumour extracts by enzyme immunoassay

Tissue homogenates were prepared in accordance with standard procedures for tumour marker measurement using EIAs, as previously described [23]. In brief, the frozen tissues were pulverized in liquid nitrogen using a Micro-Dismembrator U (B Braun Melsungen AG, Melsungen, Germany). The powder was homogenized using a tissue homogenizer (Ultra-Turrax; Janke & Kunkel, IKA-Werke, Staufen, Germany) for 20 s in three volumes of ice-cold extraction buffer. The homogenate was centrifuged at 800 *g *for 30 min at 2°C, and the resulting supernatant re-centrifuged in an ultracentrifuge (Beckman Instruments, Fullerton, CA, USA) at 100,000 *g*. The resulting supernatants (cytosols) were used for measurement of the hormone receptors (ER, progesterone receptor [PgR]), and the membrane fractions were used for EIA measurement of membrane-associated ErbB-2. ER and PgR concentrations were measured from tumour cytosolic extracts by commercial quantitative ER and PgR EIA kits (Abbott Laboratories, Abbott Park, IL, USA) using a Quantum II photometer (Abbott Laboratories, Abbott Park, IL, USA). Quality control of ER and PgR measurements was carried out in collaboration with the Receptor Biomarker Group of the European Organization for Research and Treatment of Cancer. ErbB-2 receptor levels were determined on the particulate membrane fractions of tumour extracts using a commercial monoclonal antibody EIA kit, described by Eppenberger-Castori and coworkers [23].

### Immunoassay of phosphorylated Akt level

Neither antibody used in the CLISA discriminates between Akt isoforms. The catching antibody (anti-Akt/PKB, PH domain, clone SKB1; Upstate Biotechnology, Lake Placid, NY, USA) recognizes Akt1/PKBα, Akt2/PKBβ and Akt3/PKBγ (weak to none) based on immunoblot analysis using 100 ng recombinant fusion protein for each isoform, as reported by the manufacturer. The detecting phospho-specific (S473) Akt monoclonal antibody (4E2) detects endogenous levels of Akt1 only when phosphorylated at serine-473. This antibody also recognizes Akt2 (S472) and Akt3 (S474) if they are phosphorylated at the corresponding residues, according to the information obtained from the manufacturer (Cell Signaling Technology, Inc., Beverly, MA, USA). However, 4E2 does not recognize other Akt phosphorylation sites.

S473 phosphorylated Akt levels were measured using a novel two-site CLISA. Black 96-well microtitre plates (Nunc Black MaxiSorp Surface; Nalgen Nunc International, Rochester, NY, USA) were coated with coating antibody at a concentration of 3 mg/ml of coating buffer (phosphate-buffered saline with 0.6 mmol/l EDTA) in a volume of 100 μl/well and kept at 4°C overnight. To measure P-Akt, respective tumour extracts were prepared as described above in the presence of NaF and Na_3_VO_4_. Before sample applications, the coated microtitre plates were washed five times with 200 μl/well washing buffer (25 mmol/l HEPES [pH 7.4], 300 mmol/l NaCl, 0.05% Tween-20) and then blocked for 2 hours at room temperature with 250 μl blocking buffer (25 mmol/l HEPES [pH 7.4], 300 mmol/l NaCl, 0.05% Tween-20, 3% TopBlock [Juro AG, Lucerne, Switzerland]). Blocked wells were washed five times with 200 μl washing buffer, and then 100 μl diluted tumour membrane extracts or reference material was added to the wells and incubated overnight at 4°C.

As a reference for each assay, an extract of MCF-7 cells, prepared as described above, was used. For use in the assay, MCF-7 cell extracts were sequentially diluted with sample dilution buffer (blocking buffer, proteinase inhibitor cocktail, NaF and Na_3_VO_4_) at ratios of 1×, 0.75×, 0.5×, 0.25×, 0.125× and 0.025×, and then 100 μl aliquots were incubated on each microtitre plate, together with tumour tissue extracts and negative controls (containing only dilution buffer). After incubation of the samples and reference material, wells were washed five times with 200 μl washing buffer at room temperature to eliminate unbound particles. Biotinylated detection antibody was added, followed by incubation for 2 hours at room temperature. Complexes were detected with horseradish peroxidase-conjugated streptavidin, diluted in conjugate diluents for 1 hour at room temperature. Horseradish peroxidase activity was detected using SuperSignal WestPico substrate (Pierce, Rockford, IL, USA) in a glow luminometer. The response data for diluted reference material was fitted, and the respective curve was used for the quantification of tumour extracts. The value of undiluted MCF-7 extracts was denominated as 1 U/ml.

### Quantitative real-time RT-PCR for the detection of proliferation markers

RNA was extracted using RNeasy kit (Qiagen, Hilden, Germany). Quality and quantity were checked using a Bioanalyzer 2100 (Agilent, Palo Alto, CA, USA). All genes were examined using SYBR Green I methods with Taqman 7000 (Applied-Biosystems, Foster City, CA, USA). Relative quantification (ΔΔCt) was obtained by normalization with ribosomal 18S and a standardization step with Human Universal Standard RNA (Stratagene, La Jolla, CA, USA). Quantitative real-time RT-PCR results were expressed in arbitrary units of reverse transcribed RNA (U/μg rt-RNA).

### Statistical methods

The statistical significance of the association between P-Akt and other dichotomous variables (e.g. node status) was assessed using Mann–Whitney U-test. Spearman rank correlation (r_s_) was calculated to assess associations between continuous markers (e.g. ErbB-2 or tumour size and P-Akt protein expression levels). The continuous variable function of CLISA-determined P-Akt values was first tested for prognostic significance by univariate Cox regression. A cutoff or prognostic threshold value with respect to relapse-free survival was sought by means of classification and regression tree analysis [24,25]. Survival probabilities were calculated using the Kaplan–Meier method and compared by means of log-rank analysis [26]. The Cox proportional hazards regression model was also applied over multivariate analyses, with the associated likelihood ratio test used to assess test-of-trend differences. The results of multivariate Cox regression analysis were summarized in a table and expressed as relative risk for relapse.

## Results

### Distribution of phosphorylated Akt levels and its correlation with tumour characteristics

**Figure 1:**
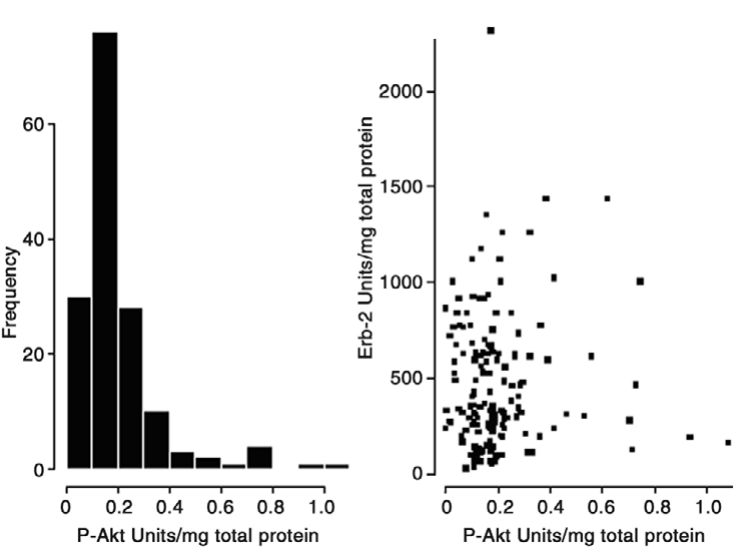
Chemiluminescence-linked immunoassay (CLISA)-quantified phosphorylated Akt (P-Akt) levels. **(a) **Histogram showing distribution of chemiluminescence-linked immunoassay (CLISA)-determined phosphorylated Akt (P-Akt) expression levels in 156 primary breast cancer samples. P-Akt levels ranged from 0 to 1.08 U/mg, with a median of 0.17 U/mg. **(b) **Scatter plot of P-Akt versus ErbB-2 expression levels. No correlation was found between the levels of P-Akt and ErbB-2.

CLISA quantified P-Akt levels have a left-tailed distribution ranging from 0 to 1.08 U/mg total protein, with a median of 0.17 U/mg (mean 0.19 U/mg; Fig. 1) and could be transformed to normality by means of the 10th root. There was no correlation between P-Akt and ErbB-2 protein expression levels. In this set of primary breast cancer samples, we did not find any significant difference in P-Akt levels with respect to nodal status, tumour size, ER status or grading, nor any correlation between P-Akt levels and the continuous variables tumour size and ER level.

### Prognostic significance of phosphorylated Akt levels

**Figure 2:**
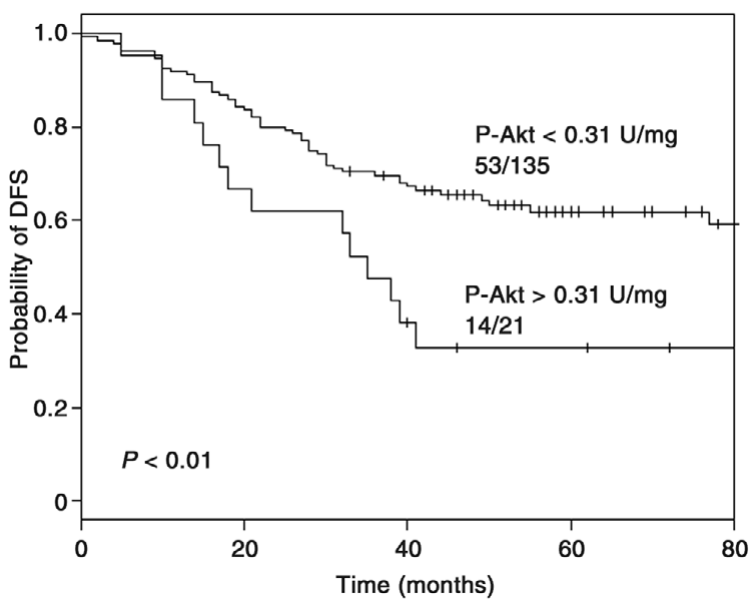
Kaplan–Meier survival curves for patients overall. The curves are stratified by phosphorylated Akt (P-Akt) levels. Patients whose tumors express high levels of P-Akt exhibit a significantly worse outcome in terms of disease-free survival (DFS; *P *< 0.01).

The prognostic value of P-Akt was investigated with respect to disease-free survival (DFS) in the patients overall (Fig. 2). Univariate Cox regression revealed a weak correlation between P-Akt levels and DFS (*P *< 0.05; likelihood ratio test). An optimal cutoff value for P-Akt (0.3 U/mg) was calculated using classification and regression tree analysis, dividing the patients into two subgroups: 21 patients (14%) patients expressed high levels of P-Akt (>0.31 U/mg total protein) and 135 patients (86%) expressed low levels of P-Akt. Subsequently, Kaplan–Meier survival curves stratified according to low and high P-Akt levels were plotted (Fig. 2). Sixty-seven per cent of patients (14 out of the 21) with high P-Akt levels relapsed, whereas only 36% (49 out of 135) with low P-Akt developed a relapse of disease within the period of observation (*P *< 0.01; log-rank test). The 5-year DFS was 33% in the high P-Akt group versus 60% in the low P-Akt group. The 5-year DFS in node-positive patients was 50% versus 68% in node-negative patients (*P *< 0.05; curves not shown).

Multivariate Cox analysis was performed including P-Akt and those additional variables that were found to have significant prognostic value in univariate Cox models (ER, ErbB-2 and node status, and tumour size and grading). In the tested multivariate model CLISA-determined elevated P-Akt level was an independent prognostic factor (*P *= 0.02), with a relative risk for breast cancer relapse of 2.09 (Table 2).

### Prognostic significance of phosphorylated Akt in ErbB2-overexpressing tumours

**Figure 3:**
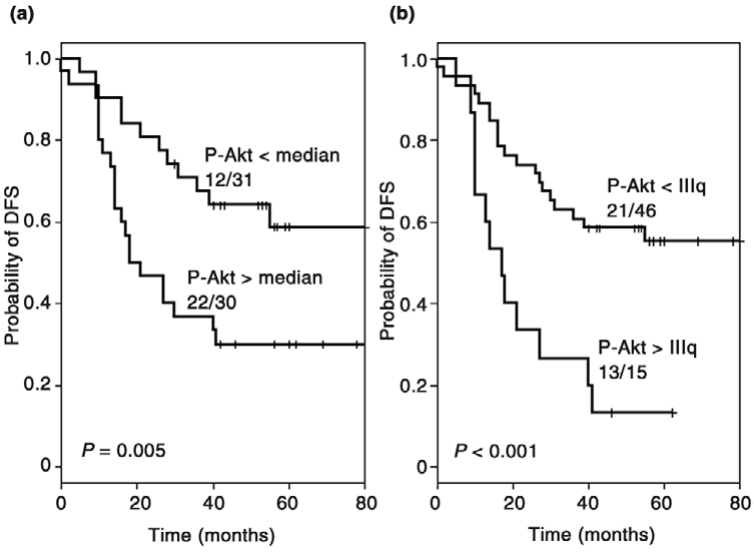
Kaplan–Meier survival curves for the subset of patients with ErbB-2 overexpressing tumours. The curves stratified by **(a) **median and **(b) **last quartile values of phosphorylated Akt (P-Akt). Patients whose tumours express high levels of P-Akt exhibit a significantly worse outcome in terms of disease-free survival (DFS; *P *≤ 0.005).

Although no correlation was found between P-Akt and ErbB-2 expression, the prognostic impact of P-Akt was greater in ErbB2-overexpressing tumours than in the samples overall. As shown in the Kaplan–Meier curves in Fig. 3a, patient prognosis decreased significantly when tumours expressed P-Akt levels higher than the median value (*P *= 0.005). This effect was even more pronounced when P-Akt levels exceeded the third quartile value (*P *< 0.001), which, together with the multivariate Cox-analysis, indicates that P-Akt has independent and additive prognostic value in combination with ErbB-2 (Fig. 3b).

### Correlation of phosphorylated Akt levels and mRNA expression of proliferation markers

**Figure 4:**
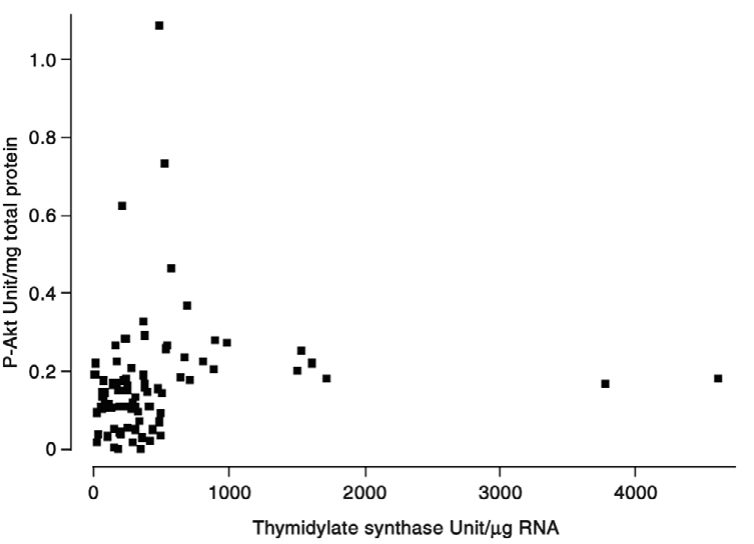
Scatter plot of phosphorylated Akt (P-Akt) versus thymidylate synthase (TS) mRNA expression. There is a good positive correlation (r_s _= 0.38; *P *< 0.001) between the two factors.

Because involvement of P-Akt has been implicated in proliferation and apoptosis, we compared the quantitative P-Akt protein levels with the quantitative mRNA expression levels of genes involved in these biological processes. Using Spearman rank correlation, P-Akt levels were found to correlate with thymidylate synthase expression levels (r_s _= 0.38; *P *< 0.001) and, to a lesser extent, with expression levels of thymidine kinase 1, survivin, topoisomerase IIα and the E2F transcription factor (Fig. 4, Table 3).

## Discussion

Correlations between elevated P-Akt and higher risk for relapsehas already been demonstrated by other investigators in certain subsets of patients, specifically patients who received adjuvant endocrine therapy [17], patients treated with radiotherapy [18] and patients with a node-negative disease [19]. Because ErbB-2 has been implicated in the activation of Akt [27], we investigated the association between P-Akt and ErbB-2 and its prognostic significance in tumours with known ErbB-2 expression levels. Our investigation re-confirmed the prognostic value of elevated P-Akt levels, and demonstrated that P-Akt expression levels are independent of other prognostic parameters, such as tumour size, grading, and node, ER and ErbB-2 status.

The lack of correlation between protein levels of ErbB-2 and P-Akt may be explained by the fact that Akt is also activated by various receptor tyrosine kinases [5-9], and by G-protein-coupled receptors [28]. Additionally, it was also observed that loss of PTEN activity is frequent in breast cancer and accompanied by increased activation of Akt [29], confirming that Akt can be activated by stimuli other than ErbB-2. The prognostic significance of P-Akt levels is increased if combined with ErbB-2 overexpression, suggesting that coactivation of Akt and ErbB-2 may have a synergistic clinical impact.

Our study is the first report on P-Akt assessed by EIA using a phospho-specific antibody in breast cancer cytosols of cryopreserved tumour samples; the technique allowed us to obtain precise and quantitative results (for review [30]). In contrast to semiquantitative immunohistochemistry data, tumour marker profiles assessed by quantitative EIA are more sensitive and reproducible. EIA tests conducted with fresh frozen tissue extracts avoid the potential antigen damage due to formalin fixation, paraffin embedding and uncontrolled storage. Furthermore, the two-site (sandwich) CLISA assay used in this investigation ensures increased specificity as compared with single-antibody assays, such as immunohistochemistry and western blotting. In addition, chemiluminometric detection guarantees high sensitivity in the detection of antigen–antibody complex.

We assayed for P-Akt in total breast tumour lysates, and not in tissue samples obtained from microdissection, both because we wished to correlate the protein expression levels of ErbB-2 and P-Akt levels directly in cells extracted from human tumour samples, and because it has been demonstrated that the activation status of Akt varies considerably in tumours of the same histotype, but not between different histotypes of the same tumour [31]. The CLISA assay used in the study was based on homogenized samples, which can include some stromal and normal tissue cells. The STB tissue samples contained at least 60% tumour cells, as observed by the pathologist. In addition, samples were previously analyzed for ErbB-2, ER and PgR using both EIA assays, as well as immunohistochemistry and/or fluorescence *in situ *hybridization. Importantly, good correlation between the assays was observed [23], suggesting that homogenization of samples does not play a crucial role in the final result. As in other assays that measure phosphorylation levels, the role played by phosphatases should not be ignored. We used phosphatase inhibitors in all steps of CLISA, as well as sample dilution. There could be some degradation before P-Akt testing, but all samples were treated identically, and the study compared relative P-Akt levels among all tumours. Reference units (U) were used in order to establish a standard curve, but not to measure absolute P-Akt levels in separate samples.

Also of interest is the positive correlation between P-Akt and mRNA expression levels of tumour proliferation markers shown in the present study. Akt is known to promote cell cycle progression by modulating the expression [32] and stabilization of cyclin D_1 _[33], which in turn activates the E2F transcription factor. Our results also reveal a significant correlation of P-Akt with E2F-1 transcription factor expression levels, as well as with genes regulated by E2F, such as thymidylate synthase, thymidine kinase 1, survivin and topoisomerase IIα.

## Conclusion

Using a highly sensitive and specific CLISA assay, we demonstrated that elevated P-Akt is a marker of poor prognosis (decreased DFS). The prognostic value of Akt phosphorylation is independent of other characteristics, including tumour size and grade, and node, ErbB-2 and ER status. In a subset of patients with ErbB-2 overexpressing tumours, we demonstrated that P-Akt levels are of particular prognostic significance. In addition, Akt phosphorylation correlated with elevated mRNA expression levels of tumour proliferation factors. Based on these findings, we suggest that P-Akt could play a predictive role with respect to Herceptin, topoisomerase IIα inhibitors and combination therapies using Akt inhibitors, which are currently in clinical trials and should primarily be assessed in patients with ErbB-2-overexpressing tumours.

## Abbreviations

CLISA = chemiluminescence-linked immunoassay; DFS = disease-free survival; EIA = enzyme immunoassay; ER = oestrogen receptor; P-Akt = phosphorylated Akt; PgR = progesterone receptor; PIP3 = phosphoinositol-3-phosphate; PKB = protein kinase B; RT-PCR = reverse transcription polymerase chain reaction; STB = Stiftung Tumorbank Basel.

## Competing interests

The author(s) delcare that they have no competing interests.

## Authors' contributions

JC carried out the development of CLISA assays, took measurements in breast cancer samples, participated in raw data analysis, participated in statistical analysis and drafted the manuscript. PU performed the statistical analysis. VV and ML carried out the RNA extraction and quantitative RT-PCR. WK participated in designing the study and participated in the raw data analysis. EW coordinated clinicians, providing tumour samples. MM helped in finalizing the manuscript. UE participated in designing the study and coordination, and helped to draft the manuscript. SE participated in coordinating the study and in statistical analysis, helped to draft the manuscript and coordinated the selection of samples from the STB. All authors read and approved the final manuscript.

## Figures and Tables

**Table 1:** Clinicopathological characteristics of the patients

Feature	Number of patients (%)
Patients enrolled	156

Age (years):	
<40	12 (8)
40–60	85 (54)
>60	59 (38)

Histology type:	
Ductal	109 (70)
Lobular	17 (11)
Other	30 (19)

Tumour size:	
T1	49 (31)
T2	90 (58)
T3-T4	17 (11)

Lymph-node status	
Node negative	66 (42)
Node positive	90 (58)

Histopathological grade	
I + II	57 (37)
III	86 (55)
Not analyzed	13 (8)

Oestrogen receptor	
Positive (>20 fmol/mg)	116 (74)
Negative (≤ 20 fmol/mg)	40 (26)

Progesterone receptor	
Positive (>20 fmol/mg)	85 (54)
Negative (≤ 20 fmol/mg)	71 (46)

**Table 2:** Univariate and multivariate Cox analysis of relapse-free survival in patients with primary breast cancer

Factor	Univariate *P*	Multivariate *P*	Relative risk for relapse	95% CI
P-Akt	0.01	0.02	2.09	1.14–3.85
Node status	0.0003	0.09	1.33	0.95–1.85
ER status	0.03	0.17	0.67	0.38–1.19
ErbB-2 status	0.002	0.04	1.73	1.02–2.94
Grading	0.03	0.06	1.57	0.98–2.50
Tumour size	0.00005	0.02	1.51	1.07–2.13

CI, confidence interval; ER, oestrogen receptor; P-Akt, phosphorylated Akt.

**Table 3:** Spearman rank correlation of quantitative P-Akt levels and quantitative mRNA expression levels of proliferation markers

Proliferation marker	r_s_	*P*
Thymidylate synthase	0.38	<0.001
Thymidine kinase 1	0.23	<0.01
Survivin	0.22	<0.01
E2F	0.22	<0.01
Topoisomerase IIα	0.19	<0.05

r_s_, Spearman correlation coefficient.
